# Oral–Gut Microbiome Axis in Crohn’s Disease: A Potential Role of Ectopic Colonization

**DOI:** 10.3390/microorganisms14040810

**Published:** 2026-04-02

**Authors:** Ceren Ozkul, Emre Duman, Engin Kocak, Yalcin Tarkan Karakan, Can Cindoruk, Odul Egritas Gurkan, Mehmet Cindoruk, Tarkan Karakan

**Affiliations:** 1 Department of Pharmaceutical Microbiology, Faculty of Pharmacy, Hacettepe University, 06100 Ankara, Turkey; emreduman@hacettepe.edu.tr; 2 Department of Analytical Chemistry, Faculty of Gulhane Pharmacy, Health Sciences University, 06018 Ankara, Turkey; engin.kocak@sbu.edu.tr; 3 Faculty of Medicine, Baskent University, 06490 Ankara, Turkey; yalcinkrkn@gmail.com; 4 Faculty of Medicine, TOBB ETU, 06560 Ankara, Turkey; ccindoruk2005@gmail.com; 5 Department of Pediatric Gastroenterology, Faculty of Medicine, Gazi University, 06560 Ankara, Turkey; odulmd2003@yahoo.com; 6 Department of Gastroenterology, Faculty of Medicine, Gazi University, 06560 Ankara, Turkey; drmehmetcindoruk@gmail.com (M.C.); tkarakan@gazi.edu.tr (T.K.)

**Keywords:** oral microbiome, gut microbiome, oral–gut axis, Crohn’s disease, IBD

## Abstract

Recently, an oral–gut communication axis has been proposed. Herein, we review clinical studies reporting differences in oral microbial communities in inflammatory bowel diseases (IBDs), with a focus on Crohn’s Disease (CD), as well as evidence from experimental models. While available studies support evidence for the direct transmission of oral-derived bacteria to gut, further work is needed to clarify whether such transmission results in stable colonization of intestinal niches and the establishment of a persistent host–microbe state that influences host physiology. To date, evidence from clinical and murine studies suggests three routes of the oral–gut axis, which in turn directly or indirectly exacerbate intestinal inflammation and contribute to IBD pathogenesis: (i) direct invasion of pathobionts through swallowing, (ii) migration of the oral pathogen activated pro-inflammatory immune cells, (iii) systemic inflammation triggered by oral pathogens such as *Porphyromonas gingivalis*. Although the role of oral microbiome in systemic diseases is becoming more apparent, sophisticated clinical and experimental studies are needed to elucidate the direct and indirect oral–gut communication mechanisms, including the contribution of oral microbial metabolites. Future directions may include evaluating the diagnostic and therapeutic potential of the oral microbiome and metabolome.

## 1. Introduction

Human microbiota is defined as the entire ecosystem of microorganisms in the body that maintains a symbiotic relationship with its host. This term encompasses microbial communities of the gastrointestinal tract, skin, urogenital system, respiratory system, conjunctiva, and oral cavity. Each of these sites harbors their own unique microbiota composition. The concept of human “microbiome” was first suggested by Lederberg and McCray in 2001, which signifies the genomic repertoire of microorganisms in health and disease states [1].

The gastrointestinal system is the primary site in the human body that supports colonization by commensal microorganisms, owing to its rich amount of fibers and nutrients, with predominantly low-oxygen conditions in the distal gut that favor anaerobic communities. The oral cavity, actually a part of gastrointestinal tract, confers an ideal environment for colonization and growth of these microorganisms, since it has ideal temperature, humidity, and nutrient status. With the variety of microorganisms (viruses, protozoa, fungi, archaea, and bacteria) that constitute the oral microbiome, this makes the oral cavity the second most abundant microbial community in the human body [2]. More than 750 species of bacteria have been identified with culture-independent methods [3]. Saliva, oral mucosa, and periodontal sites are the three compartments of the oral cavity, leading to the formation of their respective microbiomes. Since gut and oral microbial ecosystems are physically and chemically linked, an oral–gut communication axis has been proposed in both health and disease.

**Figure 1:**
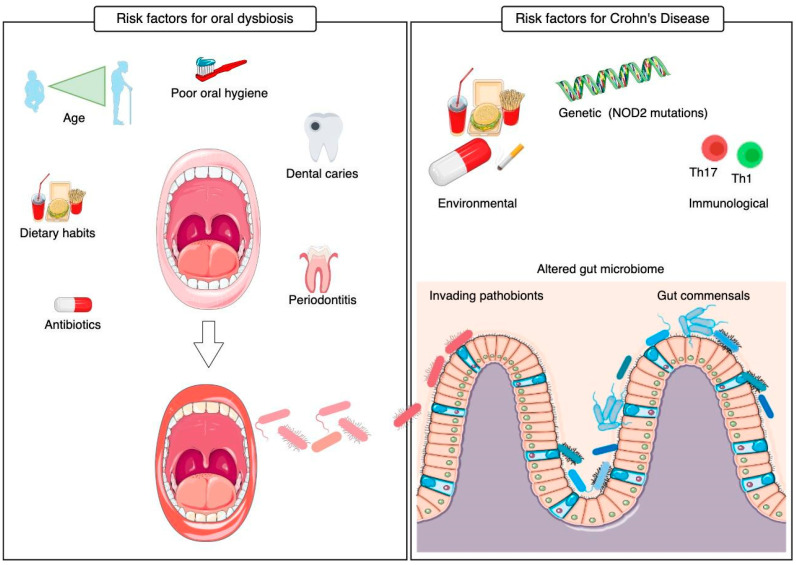
Overview of factors linked to oral dysbiosis and Crohn’s disease (CD) pathogenesis and their potential connection via oral-to-gut translocation (**left**: risk factors for oral dysbiosis; **right**: risk factors for CD).

Inflammatory bowel disease (IBD) primarily comprises Crohn’s disease (CD) and ulcerative colitis (UC). Unlike UC, CD can affect any segment of the gastrointestinal tract. Although precise etiology of CD onset and progression remains largely unknown, environmental exposures, genetic susceptibility, and abnormal mucosal immune response mainly triggered by microorganisms are widely accepted as the main factors through lifespan [4]. The dynamic interactions between developing gut microbiota and the immune system are of critical importance in early life, and perturbations in gut microbiota lead to impaired immunological tone [5], which may further lead to susceptibility to immunopathologies such as IBD [5,6,7]. In adulthood, the impaired immune response toward microbial components can contribute to chronic intestinal inflammation in genetically susceptible hosts [8]. In addition to these established environmental, genetic, and immunological risk factors, accumulating evidence suggests that oral dysbiosis may contribute to IBD pathogenesis via the oral–gut axis [9,10,11] (Figure 1).

Factors promoting oral dysbiosis include age, dietary habits, antibiotic exposure, poor oral hygiene, dental caries, and periodontitis. Oral-derived bacteria may translocate to the gut and contribute to intestinal microbial imbalance and chronic inflammation. Risk factors for CD include genetic susceptibility, environmental influences, alterations in the gut microbiome, and immune dysregulation, predominantly involving Th1 and Th17 responses. Human genetic studies have highlighted the role of pattern-recognition receptor genes in IBD susceptibility. In particular, polymorphisms in CARD15, which encodes the pattern-recognition receptor NOD2, are critical determinants of CD susceptibility [9]. NOD2 is mainly expressed in ileal Paneth cells and, to a lesser extent, in epithelial cells of the intestine and oral cavity throughout the gastrointestinal tract [10,11]. Existing work supports a mechanistic framework in which NOD2 mutations weaken Paneth cell-mediated antimicrobial defenses (including reduced α-defensin production) [12] and disrupt mucus integrity and IgA-mediated barrier functions [13,14], thereby promoting dysbiosis and loss of protective commensals [13]. In this setting, oral-derived pathobionts that reach the intestine may be more likely to persist and expand, contributing to mucosal inflammation in CD. However, direct human evidence linking NOD2 dysfunction to the stable colonization of specific oral taxa in the gut remains limited.

Microbial metabolites are largely derived from the gut microbiota, but they are also detectable in the oral cavity, urine, blood, liver, and cerebrospinal fluid [15,16,17,18]. Findings from germ-free animals extend our knowledge and emphasize the importance of microbial metabolites in host physiology [15,19]. Altered metabolome structure in periodontitis has been reported in several studies [20,21,22], but the effect of oral microbiota-derived metabolites on IBD remains unknown.

Herein, we provide a narrative review of recent clinical and experimental studies examining oral and gut microbiota in IBD. Although we discuss both major forms of IBD where relevant, the review places particular emphasis on Crohn’s disease, reflecting the available evidence and the focus of the oral–gut axis literature.

## 2. Gut Microbial Signatures of CD

Accumulating evidence suggests that the gut microbiome has an undeniable effect on the onset and progression of IBD [23]. Major alterations in gut microbial diversity and composition in treatment-naïve CD are strongly associated with disease status [24]. There are some consistent microbiome signatures to distinguish patients with IBD such as decreased bacterial diversity, reduced abundance of several communities from the phylum *Bacillota* (formerly *Firmicutes*), and increased abundance of *Actinomycetota* (formerly *Actinobacteria*) and *Pseudomonadota* (formerly *Proteobacteria*), specifically the family *Enterobacteriaceae* [25,26,27,28,29,30]. Reduced microbial diversity and resilience appear more pronounced in CD than in UC [31]. Depletion in butyrate-producing bacteria such as *Faecalibacterium prausnitzii* and *Roseburia* spp. and a bloom in the *Enterobacteriaceae* family and *Ruminococcus gnavus* have been shown in many CD cases involving adult [31,32] and pediatric age groups [33]. Butyrate, a key short-chain fatty acid, exerts anti-inflammatory effects on intestinal epithelial cells [18]. Consistently, exogenous butyrate administration has been shown to modulate Treg/Th17 balance by promoting Treg responses and IL-10, as well as to protect the colonic mucosa from chronic inflammation in experimental models of IBD [34]. Accordingly, regardless of age, depletion in butyrate producers in CD may result in a pro-inflammatory state by impairing immune homeostasis in the intestinal mucosa.

Expansion of *Enterobacteriaceae* has been consistently reported across multiple studies. In addition, an oral pathobiont *K. pneumoniae*, a member of Enterobacteriaceae, has been shown in experimental models to exacerbate inflammation in the gut, via ectopic colonization and/or through migration of pathobiont reactive immune cells to the gut [35,36].

## 3. Oral Microbial Signatures of CD

The human oral microbiome is primarily composed of *Bacillota*, *Pseudomonadota*, *Bacteroidota* (formerly *Bacteroidetes*), *Actinomycetota* (formerly *Actinobacteria*), *Fusobacteriota* (formerly *Fusobacteria*), *Spirochaetota* (formerly *Spirochaetes*), and *Saccharibacteria* (formerly TM7) at the phylum level. The expanded Human Oral Microbiome Database (e-HOMD) currently includes 836 taxa (www.homd.org; last access: 15 February 2026) [3]. At the genus level, the oral microbiome of healthy individuals is typically dominated by *Streptococcus*, *Haemophilus*, *Rothia*, *Neisseria*, and *Veillonella*, which together can represent up to 85% of the total oral microbiota [37].

Although numerous studies have examined the relationship between intestinal microbiota dysbiosis and IBD, comparatively fewer have focused on the contribution of the oral microbiota. Salivary microbiome signatures in adult CD patients have included enrichment in *Streptococcus salivarus* [38,39]. Other significantly abundant oral microbiome members in active CD include *Prevotella*, *Prevotellaceae*, *Bacteroidota*, *Bacteroidia*, *Veillonellaceae*, *Selenomanadales*, and *Negativicutes* [40]. At the phylum level, two recent studies reported an enriched oral *Actinobacteria* in CD [41], as well as in both forms of IBD [42]. More recently, a large, well-matched case–control study by DeClercq et al. [43] reported a significant reduction in salivary alpha diversity in individuals with CD compared with healthy controls. In addition to these diversity changes, genus-level analyses identified selective enrichment of oral commensals, most notably *Streptococcus* and *Veillonella*, which ranked among the most abundant members of the salivary microbiota and represented the most consistent differentially abundant signals. Although the study included both CD and UC, disease-stratified analyses revealed that these oral microbial alterations were more pronounced in CD. Consistent with these observations, Elzayat et al. [44] further reported reduced salivary microbial diversity in adult CD using full-length 16S rRNA sequencing, together with selective enrichment of several species including *Veillonella dispar*, *Megasphaera stantonii*, *Prevotella jejuni*, *Dolosigranulum pigrum*, and *Lactobacillus backii*. Collectively, these findings suggest that CD is characterized by discrete and targeted shifts in the oral microbial community rather than widespread restructuring of overall community composition.

Kelsen J. et al. [45] examined subgingival microbiota in pediatric CD and reported increased abundance of *Capnocytophaga*, *Rothia*, and TM7. While these taxa can be detected in healthy oral communities, their enrichment may be associated with disease-related shifts. Besides being a part of oral microbiome, high abundance of *Capnocytophaga* and *Rothia* have been reported in gingivitis and periodontitis, respectively [46]. Docktor et al. [47] reported decreased abundance of *Fusobacteria* and *Firmicutes* in children with CD, accompanied by reduced oral microbial diversity in CD, but not in UC. Behaviors such as thumb-sucking and nail-biting may also influence oral microbial assembly during childhood. Teich et al. [48] reported an increased risk of CD among thumb-suckers and nail-biters, a finding that may appear counterintuitive in the context of the hygiene hypothesis. One possible explanation is that these habits increase hand-to-mouth transfer of environmental microbes, including potential pathobionts, resulting in imbalanced microbial exposure rather than a broadly diverse, tolerogenic one. Such exposures may promote oral dysbiosis and potentially increase the likelihood that oral-derived taxa persist in the gut. Notably, direct mechanistic evidence linking these habits to oral-to-gut ectopic colonization in CD remains limited.

## 4. Oral–Gut Microbiome Axis: The Role of Oral Pathobionts and Ectopic Colonization

As the oral cavity is continuous with the gastrointestinal tract, the oral microbiota, which harbors more than 750 species, has been increasingly linked to gastrointestinal health [49]. Gevers et al. [24] reported that treatment-naïve, new-onset CD mucosal biopsies exhibited increased representation of taxa such as *Haemophilus*, *Veillonella*, *Neisseria*, and *Fusobacteriaceae*, which are also recognized members of the oral microbiota. Notably, identifying oral-associated taxa directly in intestinal mucosal biopsies strengthens stool-based observations and is particularly relevant to IBD, as key host–microbe interactions occur at the epithelial barrier. Among those taxa, *Haemophilus* and *Veillonella* have also been reported in the salivary microbiota of IBD patients [50]. Schmidt et al. [51] analyzed both salivary and stool samples from 470 individuals and highlighted taxa shared between saliva and stool, including *Haemophilus*, *Veillonella*, and *Streptococcus*, supporting microbial transfer along the oral–gut axis. Together, these studies provided evidence that the members of oral microbiota can ectopically colonize in the gut, not only in the pathological conditions such as IBD but also in the healthy individuals.

Moreover, Hu et al. [38] investigated both saliva and fecal samples from individuals with CD and healthy controls, and they reported that concordant gut and oral strains of *S. salivarius* might serve as a biomarker for CD. The enrichment of oral *S. salivarius* in CD is a noteworthy finding and is further supported by Goel et al. [39].

Although disease-intrinsic oral microbial signatures are not consistently observed in CD, accumulating evidence supports a context-dependent oral–gut microbiome axis. Wark et al. [52] showed that oral microbial profiles in quiescent IBD are primarily shaped by systemic and metabolic factors rather than disease activity. In contrast, Imai et al. [53] reported increased oral–gut microbiome similarity in both CD and UC, consistent with ectopic colonization of gut by oral bacteria. In line with these observations, Xu et al. [54] further showed that oral microbiota profiles could stratify UC patients with oral manifestations, with enrichment of oral genera such as *Fusobacterium*, *Oribacterium*, and *Campylobacter* being associated with poorer treatment response and increased oral–gut microbial overlap, thereby reinforcing the functional relevance of the oral–gut inflammatory axis.

*Prevotella* is also a common component of the human oral microbiome in both Western and non-Western populations, the latter being markedly more enriched [55]. Schmidt et al. [51] classified *Prevotella* as an occasionally transmitted taxa along the gastrointestinal tract. A recent metagenome-based study of fecal samples from healthy individuals reported the presence of oral-inflammation-associated *Prevotella* species in the gut, supporting the possibility of oral-to-gut microbial transfer [56]. Furthermore, an earlier study reported the high IgA-coating pattern of gut *Prevotellaceae* species, suggesting that these taxa has capacity to drive pathogenic inflammatory responses in susceptible hosts, including those with genetic risk factors such as NOD2 mutations in CD [57]. Nevertheless, further studies are needed to clarify the causative role of oral *Prevotella* species in CD progression.

An overview of studies examining the oral microbiota in IBD is presented in Table 1. Findings specific to CD are highlighted, where a distinct profile for CD is available; unless otherwise indicated, results reflect IBD cohorts overall. It should be noted that majority of the clinical studies summarized in Table 1 include subjects from Asian populations [50,58,59]. Accordingly, oral and gut microbial signatures associated with IBD may exhibit a geographical pattern. For example, Hu et al. [38] highlighted the geographical/ethnic differences, reporting that fecal enrichment of *Clostridium nexile* was not observed among CD subjects of European descent [38].

A recent report highlighted that there is no evidence for stable colonization of oral-derived bacteria in the colon [60]. Indeed, colonization by the oral microbes can be persistent or transient. If it is transient, it cannot be classified as a true host–microbe state influencing disease status. Further sophisticated studies are needed to enlighten colonization capacity of oral-derived bacteria along the gastrointestinal track. Li et al. [61] constructed a human oral microbiota-associated mouse model by transplanting human saliva into germ-free (GF) mice. Their results indicated the ecological invasion of oral bacteria, predominantly *Porphyromonas*, largely in the small intestines, with lower levels in the colon. This result indicates that oral-derived taxa may preferentially persist in the small intestine under certain conditions and may contribute to shaping small-intestinal microbial communities.

It should be noted that identifying the same taxa in both oral and stool samples could not precisely prove the oral–gut axis and its contribution to health and disease. Clinical studies support a potential role for oral-to-gut transmission in IBD, but causal inference remains limited. Microbial Source Tracking tools using a Bayesian model may provide, to some extent, a useful insight into clinical studies [61,62]. However, mechanistic insight has largely come from experimental models, including germ-free mice inoculated with human saliva or oral isolates [36]. Murine studies do not negate the importance of genetic susceptibility in disease onset, but they highlight how dysbiosis can exacerbate intestinal inflammation [36,63]. Importantly, human-to-mouse transplantation studies have inherent limitations including incomplete and strain-dependent engraftment and cross-species differences, as GF intestines can exhibit developmental and immunological differences that may bias colonization dynamics and host responses [64].

**Figure 2:**
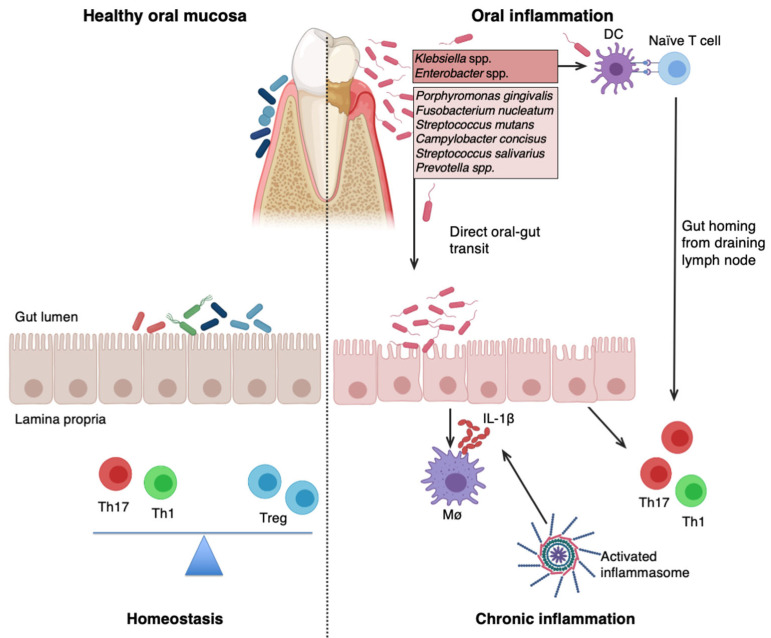
Protective and pathogenic role of oral–gut microbiota axis in IBD (adapted from Kitamoto et al. [36]). Healthy oral mucosa (**left-hand side**) comprises the non-pathogenic oral (salivary and mucosal) microbial community. This indigenous microbial community and their fermentation products can protect oral mucosa from pathogen colonization, accordingly protecting intestines from exogenous pathogen colonization. Commensal microbiota in gut, together with a subset of pathobionts, maintain gut homeostasis through Th17/Treg balance. Th1 cells are also known to increase in the lamina propria of CD patients. Together, balance in immune response and commensal microbiota protect the gut from inflammation. Oral pathobiont colonization (**right-hand side**) may lead to oral inflammation. *Klebsiella* spp. and *Enterobacter* spp. can directly translocate and ectopically colonize the gut. Secondly, oral pathobiont-activated dendritic cells promote the differentiation of CD4+ T cells into pro-inflammatory T cell subsets, including Th17. This T cell subset can transmigrate from the oral mucosa to the gut, and it is further activated by pathobionts. *Klebsiella pneumoniae* has capability to strongly induce Th1 response, which may contribute to CD pathogenesis. Other potential pathobionts include *Porphyromonas gingivalis*, *Fusobacterium nucleatum*, *Streptococcus mutans*, *Campylobacter concisus*, *Streptococcus salivarius,* and *Prevotella* spp. Direct migration of activated Th17 cells by these bacteria has not been previously shown. Thus, their direct oral–gut transit may be another mechanism for intestinal inflammation. Ectopic colonization of oral pathobionts may destroy the intestinal epithelial barrier (1), activating lamina propria macrophages and inflammasomes, which then leads to excessive IL-1β secretion (2) and triggers inflammation. A dysbiosis state caused by loss of commensals, accumulation of gut pathobionts, and ectopic colonization of oral pathobionts is induced, which leads to chronic inflammation involving hyperactivation of Th1 and Th17 cells. In this state, suppression of pathobionts by other microbiota members is diminished, as induction regulatory immune response involving Treg cells is also disrupted due to the imbalanced microbiota.

A recent study by Kitamato et al. [36] provides mechanistic insight for periodontal inflammation to colitis. They reported that periodontitis induces expansion of oral pathobionts, including *Klebsiella* spp. and *Enterobacter* spp., in the oral mucosa. Using a murine model of ligature-induced periodontitis, they showed that periodontal inflammation exacerbated gut inflammation in DSS-induced colitis compared with non-ligature DSS controls, accompanied by increased ectopic colonization of *Klebsiella/Enterobacter* in the lower gastrointestinal tract. They proposed two mechanisms for pathobiont-driven colitis. First, ectopic colonization of gut by oral pathobionts exacerbate colitis by activating lamina propria macrophages, leading to excessive IL-1β secretion. Second, oral pathobiont-reactive Th17 cells migrates to the inflamed gut and can be activated by ectopic pathobionts in the gut, contributing to exacerbation of inflammation [36]. Figure 2 summarizes these two proposed mechanisms for the effect of oral pathobionts on gut inflammation and highlights additional candidate oral pathobionts suggested by experimental or clinical studies in the literature.

Findings from the Kitamato study may also provide an explanation for higher prevalence of gum disease and poor oral health in IBD patients, a fact that also shows the causal link between IBD and periodontal disease [65]. For example, CD patients have been reported to exhibit a higher prevalence of periodontitis than healthy controls [66]. Other oral manifestations of IBD are well-described in the literature, especially for CD, including mucosal tags, cobblestoning, and deep linear ulcerations with vertical fissures [67,68].

Furthermore, *K. pneumoniae* has attracted particular interest in CD. Atarashi et al. [35] transplanted saliva samples from two CD patients into germ-free mice and observed a significant increase in the amount of Th_1_ cells in the intestinal lamina propria along with an increased ectopic colonization of *K. pneumoniae* in fecal samples. Moreover, oral administration of isolated *K. pneumoniae* strain was sufficient to induce TH_1_ cells. Taken together, these experimental studies provide strong evidence for the colitogenic effects of ectopically colonized oral pathobionts in IBD.

Other studies have also suggested that severity of IBD may be exacerbated by specific oral bacteria, including *Porphyromonas gingivalis* [69,70], *Streptococcus mutans* [71], *Fusobacterium nucleatum* [72], *Campylobacter concisus* [73], *Klebsiella* spp. [35,36], and *Streptococcus salivarius* [38], which may also show ectopic colonization, as shown in Figure 2.

*P. gingivalis* is a keystone pathogen in chronic periodontitis and has been implicated in periodontal manifestations observed in CD patients [69]. A recent study evaluated the colitogenic potential of several oral pathogens in a murine model of colitis and reported that *P. gingivalis* had high capability to exacerbate colitis [70]. Gingipains secreted by *P. gingivalis* can degrade host proteins and cytokines, and it is suggested as a potential contributor in IBD pathogenesis [70]. *P. gingivalis* is likely to contribute to UC progression rather than CD, as it can induce a strong Th2 response [70,74]. Consistently, transfer of salivary microbiota from periodontitis subjects exacerbated inflammation and worsened colitis in specific-pathogen-free (SPF) mice, with a Th2-dominant immune response [75]. Although direct evidence for transmission and persistence of oral *P. gingivalis* in CD patients is limited, this bacterium may represent one of the clear connections between oral and gut dysbiosis, considering its capacity to increase inflammation [70]. From an ecological perspective, *P. gingivalis* displays high metabolic plasticity that allows high adaptation to different niches below the gum line [76], as its ecological invasion ability has been previously shown in murine models [61]. Of note, as a keystone pathogen in periodontitis, *P. gingivalis* can also induce systemic inflammation, which in turn indirectly contributes to exacerbation of intestinal inflammation, which is considered as another mechanism of the oral–gut axis [77].

## 5. Th-17 Pathway in the Oral–Gut Axis in CD

Across mucosal surfaces, Th17 cells are critical regulators of barrier immunity. Amplification and dysregulation of IL-17-secreting cells in disease settings has been linked to immunopathology [78]. Unlike other barrier sites, such as the skin and lower GI tract, Th17 cells may arise at the oral mucosa independently of commensal colonization. Indeed, ongoing damage through mastication is a unique tissue-specific trigger for the development of homeostatic Th17 cells in oral tissues [79]. The Th17 lineage, acting largely through IL -17, has been elegantly shown in animal models to confer a dominant protective response to oral candidiasis through neutrophil recruitment and induction of antimicrobial factors [80]. Inflammatory oral diseases, such as periodontitis, can be exacerbated by pathobiont-responsive Th17 cells within a gut–mouth immune axis, and these responses may be influenced by the intestinal microbiome [81]. Accordingly, targeting the migration of pathobiont-responsive Th17 cells to the oral tissues can be a potential therapeutical target to block the pathogenic gut–mouth axis in the development of inflammatory oral diseases.

Lee et al. [82] analyzed the data from Crohn’s Disease Viral and Microbial Metagenome Project (PRJEB3206) and highlighted the increased levels of *Porphyromonadaceae* in patients with CD. They also showed that *P. gingivalis* challenge in experimental colitis increased disease severity. It has been proposed that *P. gingivalis* derived from periodontitis may translocate to the intestine and induce an abnormal immune response characterized by elevating the Th17/Treg ratio [83]. Although these findings are derived primarily from experimental colitis models, rather than direct evidence in CD, they provide strong evidence for the dual role of ectopic colonization and Th17 response in CD pathogenesis.

## 6. Microbiota-Derived Metabolites

With the development of metabolomics approaches, oral metabolites have increasingly been associated with periodontal [22] and systemic diseases [84]. Well-characterized microbial metabolites such as short-chain fatty acids have established roles in gut health [34,85,86]. However, whether a similar function for these metabolites in the oral cavity exists or not is largely unknown. Therefore, studies profiling the salivary and periodontal metabolome in CD are needed.

To date, the role of oral microbial metabolites in CD is not supported by direct evidence. Conceptually, oral metabolites are most likely to exert their primary biological effects locally within the oral cavity, where they can modulate mucosal inflammation and ecological balance of the oral microbiome. Given dilution, absorption, and metabolic transformation during gastrointestinal transit, direct delivery of intact oral-derived metabolites to the colon at biologically meaningful concentrations is expected to be limited. Accordingly, potential downstream gut effects are more likely indirect. In this context, several indirect pathways can be hypothesized: (i) oral microbial metabolites can trigger to trigger local inflammation and exacerbate periodontitis [22], which may further perturb microbial communities; (ii) transmission of oral microbiome members that reach the intestine may contribute to alterations in the gut microbiome and metabolome structure; (iii) translocation of oral microbiome members and their metabolites to the bloodstream, due to poor oral hygiene, subgingival irrigation, and flossing [87], may influence systemic inflammation. The oral microbial metabolites most likely to enter the bloodstream include short-chain fatty acids [88], trimethylamine N-oxide (TMAO) [89], indole and tryptophan derivatives [90], bile acid metabolites [91], and lipopolysaccharides (LPS) [92]. Moreover, these oral microbes and their by-products in the bloodstream may use a hematogenous route to reach the colon [93]. Therefore, both direct invasion and hematogenous transmission of oral microbiota and their metabolites may represent a significant component of the oral–gut axis. Given the lack of direct evidence for oral–gut metabolic axis, and considering the potential mechanisms, we will discuss this part focusing on oral metabolome profile in periodontal diseases and gut metabolome in CD.

Microbial metabolites are important modulators of host physiology and mediators of microbe–host crosstalk. In periodontal disease, metabolomic profiling has consistently demonstrated local enrichment of inflammatory-associated metabolites [20]. For example, Gawron et al. [22] reported increased lactic acid in chronic periodontitis patients with higher concentrations at *P. gingivalis*-positive sites, and they observed that acetone, which is closely associated with lactic acid, increased with inflammation. Similarly, Garcia-Villaescua et al. [21] reported increased lactic acid at early onset of periodontitis. Although lactate is a well-established circulating metabolite with systemic signaling effects, current evidence primarily supports elevated lactate within oral biofluids and periodontal niches, whereas direct evidence that oral microbiota-derived lactate enters systemic circulation or directly contributes to gut inflammation remains insufficient. Barnes et al. [94] used LC/MS based metabolomics and reported increases in multiple energy metabolites, amino acids, fatty acids, and carnitine derivatives in periodontitis. Together, these studies suggest that dynamic metabolome structure may reflect the inflammation status and disease onset and can be used as a potential biomarker in inflammatory diseases. However, further studies with are needed to clarify whether and how oral microbiota-derived metabolites contribute to inflammation and IBD-relevant pathways.

## 7. Conclusions

The mouth and gut are in constant communication in part through the continuous swallowing of oral bacteria. Accordingly, an oral–gut communication axis has been recently proposed, and a growing body of evidence in the literature suggests the direct and indirect role of periodontal pathogens in gut microbial alterations, abnormal immune response, and intestinal barrier integrity.

This review summarizes clinical and experimental evidence supporting a potential role of the oral–gut axis in CD. Clinical studies have reported oral dysbiosis in CD, although findings are not fully consistent across cohorts. Notably, inconsistent results may reflect the role of dysbiotic microbial patterns, rather than changes in a specific taxonomic group in disease pathogenesis. In addition, the results from clinical studies should be interpreted with caution due to the relatively small sample sizes, the use of anti-inflammatory medications in most of the cases, and well-characterized geographical/ethnic differences. Although there have been limited studies in CD, the periodontal keystone pathogen *P. gingivalis* has been shown to successfully transmit through the GI tract, with high adaptation capabilities to different niches [61,76]. Although the direct transmission of oral-derived microbes to the gut has been demonstrated, further sophisticated studies are needed to clarify whether such transmission results in stable intestinal colonization and maintains a true host–microbe state by directly affecting host physiology.

Murine studies provide more evidence for the potential pathogenic role of oral microbiota in IBD. *Klebsiella pneumoniae* are likely to be closely related to oral pathobionts, particularly in CD pathogenesis, considering its capability to induce Th1 response in the gut [35]. Oral pathobionts have ability to trigger inflammation either by direct translocation into the gut or by inducing pro-inflammatory T cells in oral mucosa, which can further migrate to gut from draining lymph node [36].

Although there is scarce evidence, the findings from murine studies and clinical studies demonstrating the common taxa in oral and fecal samples from CD patients indicate that oral dysbiosis and poor oral hygiene are likely to be important risk factors for CD, along with the environmental, immunological, and genetic factors.

Beyond microbial transfer, oral microbe-derived metabolites may also contribute to the oral–gut axis, although current evidence remains limited. To date, oxidative status markers, inflammatory cytokines, and microRNAs have been proposed as salivary biomarkers of IBD [95]. Potential use of oral microbial metabolites for therapeutic applications or as diagnostic biomarker needs further investigation.

## 8. Key Points

Recent studies provide evidence for mechanisms of the role of the oral–gut axis in the onset and progression of CD:

- Periodontal pathogens such as *P. gingivalis* can induce local and systemic inflammation, which may indirectly exacerbate intestinal inflammation.
- Ectopic colonization of pathobionts such as *Klebsiella* may contribute to onset and progression of CD.
- Oral microbiota-derived, pathobiont-reactive T cells can migrate to the gut, leading to increased inflammation.

## Figures and Tables

**Table 1:** Clinical studies on oral and oral–gut microbial community differences in IBD.

Study Design	Sample Size	Sample	Main Findings	Reference
Adult IBD	CD (*n* = 31) UC (*n* = 26) HC (*n* = 24)	Upper gumline (gingival/tooth interface)	No significant differences in α/β diversity between IBD in remission and healthy controls. Oral diversity did not associate with disease activity, fecal calprotectin, oral extraintestinal manifestations, or future flare risk. Differentially abundant *Campylobacter*, *Fusobacterium*, *Veillonella*, and *Prevotella* in IBD patients using biological therapy compared to those who were not (LEfSe; *p* < 0.05, LDA > 3.5). Biologic therapy associated with lower α-diversity (*p* = 0.01, CI 0.58–0.73) and altered composition. Higher fasting insulin levels correlated with increased oral microbial alpha diversity (richness r = 0.47, adj-*p* < 0.01) and beta diversity (Pseudo-F = 2.05, *p* = 0.02).	Wark et al. [52]
Adult IBD	CD (*n* = 65) and match HC (*n* = 65), UC (*n* = 64) and match HC (*n* = 64), Both CD and UC (*n* = 31) and match HC (*n* = 31)	Saliva	Alpha diversity significantly lower in IBD participants, especially among females (*p* ≤ 0.05); beta-diversity differences were not significant after covariate adjustment. Four genera—*Fusobacterium*, *Ottowia*, *Ruminococcaceae*_[G-1], and *Tannerella*—were differentially abundant in CD when compared to HC (ANCOM-BC, q = 0.067, 0.078, 0.024, 0.004, respectively) *Veillonella* and *Streptococcus* showed higher relative abundance in IBD cases than in controls (25% vs. 22% and 14% vs. 12%, respectively).	DeClercq et al. [43]
Adult IBD	CD (*n* = 18), UC (*n* = 42), HC (*n* = 45)	Saliva and fecal	Salivary community composition differed between IBD and healthy controls. In CD, *Fusobacterium* and *Streptococcus* were enriched vs. controls, whereas *Enterobacteriaceae* and *Campylobacter* were relatively higher in HC (LEfSe; *p* < 0.05, LDA > 2). Stool samples showed increased representation of oral-associated taxa in IBD when compared to healthy controls (*Prevotella*, *Porphyromonadaceae*, *Neisseria*, and *Veillonella* in UC; *Prevotella*, *Porphyromonadaceae*, and *Atopobium* in CD) (LEfSe; *p* < 0.05, LDA > 2). CD and UC gut microbiome was significantly more similar to the oral microbiome than in the HC (Jaccard; *p* < 0.01).	Imai et al. [53]
Adult UC	OU (*n* = 18) UC (*n* = 37) UC_OU (*n* = 17) HC (*n* = 28)	Saliva, buccal, and fecal	UC patients with oral ulcers exhibited significantly distinct oral microbiome profiles and poorer response to 5-ASA therapy. *Fusobacterium*, *Oribacterium*, and *Campylobacter* were enriched in non-responders. Salivary profiles (saliva > buccal > fecal) best discriminated/stratified UC patients with oral manifestations and were linked to IL-17/Th17-related immune activation signatures.	Xu et al. [54]
Adult CD	CD (*n* = 40) HC (*n* = 40)	Saliva	Salivary alpha diversity was significantly reduced in CD compared with healthy controls in terms of observed species, Chao 1 and ACE (*p* values: 0.049, 0.022, and 0.048, respectively). Five bacterial species (*Veillonella dispar*, *Megasphaera stantonii*, *Prevotella jejuni*, *Dolosigranulum pigrum*, and *Lactobacillus backii*) were significantly enriched in CD (LEfSe; *p* < 0.05, LDA > 2). Monoclonal antibody therapy was associated with *Simonsiella muelleri*, while combination therapy promoted enrichment of pathogenic Proteobacteria (*Escherichia coli*, *Salmonella enterica*, *K. pneumoniae*, *Pseudomonas aeruginosa*, and *Enterobacter cloacae*). Salivary microbial diversity negatively correlated with inflammatory markers, particularly calprotectin.	Elzayat et al. [44]
Adult CD	CD (*n* = 25) HC (*n* = 25)	Saliva and fecal	*Streptococcus salivarius* was enriched in both saliva and stool of CD patients; this concordance was more frequent in individuals with a higher Crohn’s Disease Activity Index (*p* = 0.012) and active disease status (*p* = 0.016). *Faecalibacterium prausnitzii* (*p* = 0.010), *Roseburia inulinivorans* (*p* = 0.010), and *Alistipes senegalensis* (*p* = 0.029) were reduced in the gut microbiota of CD patients. *Clostridium nexile* (*p* = 0.038) and *Ruminococcus gnavus* (*p* = 0.043) were enriched in the gut microbiota of CD patients.	Hu et al. [41]
Adult IBD	CD (*n* = 30) UC (*n* = 17) HC (*n* = 18)	Saliva, tongue, buccal, plaque and fecal	Oral microbiome dysbiosis in IBD was site- and taxon-specific and was more pronounced in plaque samples (Bray–Curtis; PERMANOVA *p* < 0.05). Oral microbiota could distinguish IBD from controls, with saliva performing best (random forest; mean AUC 0.73 for saliva vs. 0.67 for stool). Alpha diversity was significantly reduced in fecal samples (Chao1; *p* < 0.001) but not in salivary samples (Chao1; *p* = 0.082). *Actinobacteria*, *Bacteroidetes*, and *Spirochaetes* were reported to be higher across oral sites; however, these differences were not statistically significant.	Somineni et al. [42]
Adult OFG and CD	Only OFG (*n* = 78) OFG and CD (*n* = 40) Only CD (*n* = 97) HC (*n* = 46)	Saliva	Increased *Streptococcus salivarius* in OFG or CD compared with HC (AMOVA; *p* < 0.001). Microbial community (Beta diversity) in OFG + CD group was significantly different from the HC group.	Goel et al. [39]
Adult CD	CD-Active (*n* = 29) CD-Remission (*n* = 31) HC (*n* = 31)	Saliva	Salivary alpha diversity was lower in active CD than in remission and healthy controls (Chao1: active vs. remission *p* = 0.0015; active vs. healthy *p* = 0.00011). Significantly different unweighted and weighted UniFrac difference between all groups (Adonis; *p* = 0.001). 30 differentially abundant taxa in active CD, including *Prevotella*, *Prevotellaceae*, *Bacteroidetes*, *Bacteroidia*, *Veillonellaceae*, *Selenomanadales*, and *Negativicutes* (LEfSe; *p* < 0.05, LDA > 4). In the genus level, *Pedobacter*, *Megasphaera*, *Salmonella*, *Clostridium XI*, *Solobacterium*, *Oribacterium*, *Mogibacterium*, *Atophobium*, and *Lachnoanaerobaculum* were enriched in active CD (LEfSe; *p* < 0.05, LDA > 4). Depletion of *Neisseria*, *Haemophilus*, *Fusobacterium*, and *Porphyromonas* in active CD (LEfSe; *p* < 0.05, LDA > 2).	Zhang et al. [40]
Adult CD with oral manifestations	CD (*n* = 24) HC (*n* = 25)	Saliva	Oral microbiome dysbiosis in CD. Increased levels of *Actinobacteria* and *Proteobacteria* in saliva microbiome of CD patients. Species-level multivariable modeling identified 32 differentially abundant species (FDR-controlled,), as well as a saliva-based random forest model discriminated CD from controls with AUROC ≈ 0.84.	Hu et al. [41]
Adult IBD	CD (*n* = 13) UC (*n* = 54) HC (*n* = 25)	Saliva	No significant difference in microbial richness across groups. Bray–Curtis distances revealed a significantly reduced degree of conservation in the bacterial community structure of CD (*p* < 0.001). CD saliva showed enrichment of *Veillonellaceae (Veillonella)* and depletion of *Neisseriaceae (Neisseria)* and *Haemophilus.*	Xun et al. [58]
Adult IBD	CD (*n* = 21) UC (*n* = 14) HC (*n* = 24)	Saliva	Salivary richness/evenness did not differ between groups; however, *Neisseria* and *Gemella* were enriched in CD versus HC (*p* < 0.01 and *p* = 0.001, respectively). Compared with healthy controls, CD showed increased *Bacteroidetes*, *Veillonella*, and *Prevotella* (*p* < 0.01) and decreased Streptococcus and Haemophilus (*p* < 0.05). Salivary immune markers were altered in IBD (including CD), with higher IgA and lower lysozyme (*p* < 0.01); IL-1β correlated negatively with *Streptococcus* (r = −0.54, *p* < 0.001) and positively with *Prevotella* (r = 0.58, *p* < 0.001).	Said et al. [50]
Adult IBD	CD (*n* = 12) UC (*n* = 10) HC (*n* = 8)	Saliva	Community structure differed between IBD and controls (weighted UniFrac PCoA; Adonis *p* < 0.05) Several taxa were significantly increased in IBD saliva, including *Saccharibacteria (TM7)*, *Absconditabacteria (SR1)*, *Leptotrichia*, *Prevotella*, *Bulleidia*, and *Atopobium* (*p* < 0.05); *SR1* was higher in CD than in healthy controls (*p* < 0.05). Oral taxa were linked to inflammation markers: *SR1* correlated positively with fecal calprotectin (r = 0.370, *p* < 0.05), and multiple genera showed significant correlations with WBC/ESR/CRP; a ROC panel (*TM7* + *SR1* + *Bulleidia*) discriminated CD from controls with AUC = 0.948 (*p* < 0.001).	Qi et al. [59]
Pediatric IBD	CD (*n* = 40); UC (*n* = 31); HC (*n* = 43)	Tongue and buccal mucosal brushings	Tongue samples from pediatric CD showed reduced overall oral diversity versus healthy controls (Shannon: *p* = 0.015), driven by significant reductions in Fusobacteria (*p* < 0.0002) and Firmicutes (*p* = 0.022). No significant change in Shannon diversity was observed in tongue samples from UC patients (*p* = 0.418). Buccal mucosa samples in the CD cohort showed a trend toward reduced Shannon diversity (*p* = 0.091). In UC tongue samples, decreased *Fusobacteria* (*p* = 0.006) and increased *Spirochaetes* (*p* = 0.006), *Synergistetes* (*p* = 0.009), and *Bacteroidetes* (*p* = 0.030).	Docktor et al. [47]
Pediatric CD—Before and after antibiotics	Discovery cohort: CD (*n* = 35) HC (*n* = 43) Validation cohort: CD (*n* = 43) HC (*n* = 31)	Subgingival plaque samples	Subgingival community structure differed between pediatric CD and healthy controls at baseline (unweighted UniFrac: discovery *p* = 0.025, R^2^ = 0.027; validation *p* = 0.018, R^2^ = 0.025; weighted UniFrac: discovery *p* = 0.036, validation trend *p* = 0.09). Eleven candidate genera were replicated in the validation cohort; *Capnocytophaga*, *Rothia*, and *TM7 (Saccharibacteria)* were consistently enriched in CD vs. controls (validation: *p* = 0.001, 0.001, 0.004, respectively; increased in CD). Antibiotic exposure was associated with reduced abundance of multiple genera (e.g., *Alloprevotella*, *Fusobacterium*, *Porphyromonas*, *Prevotella*, *Selenomonas*, *Veillonella*; all *p* ≤ 0.005).	Kelsen et al. [45]

Abbreviations: OFG: orofacial granulomatosis; CD: Crohn’s disease; UC: ulcerative colitis; OU: oral ulcers; UC_OU: ulcerative colitis patients with oral ulcers; HC: healthy control.

## Data Availability

No new data were created or analyzed in this study. Data sharing is not applicable to this article.

## Abbreviations

The following abbreviations are used in this manuscript:

IBD	Inflammatory bowel disease
CD	Crohn’s disease
UC	Ulcerative colitis
OFG	Orofacial granulomatosis
OU	Oral ulcers
UC_OU	Ulcerative colitis patients with oral ulcers
HC	Healthy control
GF	Germ-free
SPF	Specific-pathogen-free
LPS	Lipopolysaccharides
TMAO	Trimethylamine N-oxide
